# Adaptation and post-adaptation effects of haptic forces on locomotion in healthy young adults

**DOI:** 10.1186/s12984-018-0364-0

**Published:** 2018-03-13

**Authors:** Gianluca U. Sorrento, Philippe S. Archambault, Joyce Fung

**Affiliations:** 1 School of Physical & Occupational Therapy, McGill University, Laval, Québec Canada; 2 Centre for Interdisciplinary Research in Rehabilitation of Greater Montreal (CRIR), Jewish Rehabilitation Hospital, site of CISSS-Laval, Laval, Québec Canada

**Keywords:** Gait, Locomotor adaptation, Haptics, Virtual reality, Sensorimotor integration

## Abstract

**Background:**

Developing rehabilitation strategies to improve functional walking and postural control in patients is a priority for rehabilitation clinicians and researchers alike. One possible strategy is the use of sensory modalities to elicit adaptive locomotor gait patterns. This study aimed to explore to what extent haptic inputs, in the form of forward-leading tensile forces delivered to the hand, compared to no force, may lead to adaptation and post-adaptation effects on gait parameters, during and after the haptic exposure, respectively.

**Methods:**

Thirteen healthy young individuals were recruited for this study. We developed an innovative system combining virtual reality and haptic tensile forces in the direction of locomotion to simulate walking with a dog. A robotic arm generated forces via an adapted leash to the participant’s hand while they walked on a self-paced treadmill immersed in a virtual environment with scene progression synchronized to the treadmill.

**Results:**

All participants showed significant increases in instantaneous gait velocity and stride length, with accompanying decreases in double-limb support time (*p* < 0.05) when walking with a haptic tensile force of either 10 or 20 N, relative to pre-force epoch levels, indicating an adaptation effect. When the 10 or 20 N force was removed, gait measures generally remained changed relative to baseline pre-force levels (*p* < 0.05), providing evidence of a post-adaptation effect.

**Conclusions:**

Changes in spatiotemporal outcomes provide evidence that both adaptation and post-adaptation effects were present in response to the application and removal of a haptic force. Future studies will investigate whether similar changes in elderly and post-stroke populations can be actualized during steady-state walking.

## Background

In physical rehabilitation, independent walking is an important outcome for reintegration into the community [1–3]. To achieve this, both gait and postural reactions must be performed in a safe and functional manner. This dynamic stability depends not only on physical but environmental and task dependent circumstances [4, 5]. Safely crossing a busy street intersection, for example, involves all these factors. Maintaining dynamic stability requires responses that are both proactive for adapting to the environment and reactive to perturbations and obstacles that may be present in the surroundings [6, 7]. This dynamic stability can be achieved when the individual’s gait and posture are recovered well enough to produce the required gait speed [8–10], symmetry and postural reactions [11] needed to walk in an effective and safe manner to avoid collisions and falls [7]. These spatiotemporal parameters help predict falling in populations with reduced mobility, such as older adults and stroke survivors [8]. In fact, these two populations have alarmingly high incidences of falling: annually, falls can occur in 35% - 45% of individuals older than 65 years [12] and increases with health risk factors [13], while other studies report a similar incidence in post-stroke populations [14, 15].

Researchers and clinicians are addressing this challenge with strategies that focus on training functional gait adaptations [16, 17]. These adaptations, in the context of procedural learning, occur as an error-driven learning process that alters well-established motor patterns in the brain [16, 18, 19]. This process is useful when an individual must adjust to different physical, environmental and task demands. In the context of gait, there is evidence to suggest such adaptations may be driven to restore the dynamic stability [20] necessary to safely reintegrate individuals into community dwelling. Two important adaptation strategies are relevant to the present study. One involves creating spatiotemporal constraints, such as modified walking surfaces [21–23]. The other employs multisensory stimuli [24–26], such as somatosensory cues [27–29].

Perhaps more significant to functional recovery is the extent to which locomotor adaptations can be prolonged after the exposure is removed [30]. This particular retention can be conceived as a post-adaptation, or a ‘carry-over’ response in locomotion. Adaptation and post-adaptation, once used to describe the ‘broken elevator effect’ by Reynolds and Bronstein (2003), have been investigated in subsequent studies [21, 31, 32]. Researchers have often used the split-belt treadmill to investigate this phenomenon. Essentially, the split-belt treadmill has two separate belts that can move both legs in tandem and at different speeds or directions. These changes can constrain or change bilateral locomotion. For example, Choi et al. (2007) suggest that the functional circuitry of both legs can be trained separately [33], potentially mitigating the effects of asymmetric gait [34]. Keeping with this approach, researchers examined post-adaptation changes in gait on a split-belt treadmill for healthy individuals and for those with neurological deficits [22, 35]. For example, Reisman et al. (2007) reported a reduction in spatiotemporal discrepancies between the paretic leg and non-paretic leg in post-stroke individuals during post-adaptation responses [22]. These changes were found after participants adapted to different treadmill speeds [21, 22]. This post-adaptation has also been successfully transferred to overground walking [36, 37]. Yet, it is less clear as to what extent this strategy could induce longer lasting post-adaptation effects [30]. Researchers believe that access to adapted motor patterns remains available in the CNS, despite transient effects and eventual unlearning [38]. Hence, the potential for lasting post-adaptations is possible, particularly with repeated exposure to a given stimulus (e.g. as part of a rehabilitation intervention).

Haptic cues may constitute another approach for retraining dynamic gait stability. For example, there is evidence that light finger contact with an earth-fixed object during locomotion can have an attenuating effect on sway and reduce postural deviations [28, 39]. Researchers also reported similar effects of light finger contact with a static external object in terms of greater postural stability in the lower limb [29]. Moreover, Fung & Perez (2011) reported that the use of an instrumented cane for both post-stroke and healthy participants holds ecological validity with the potential of carry over to overground walking [40]. Yet another strategy for providing helpful haptic cues to chronic stroke individuals is the use of a specially trained rehabilitation dog. In one study, chronic stroke survivors increased gait velocity and decreased gait variability when walking with a trained rehabilitation guide dog [41]. In light of these previous studies, the current study uses an innovative and novel approach combining haptic tensile forces in the form of a ‘virtual leash’ within a virtual environment in order to investigate the possibility of adaptation and post-adaptation effects in human gait.

Specifically, the research question is: in young healthy individuals, to what extent does a haptic tensile force applied to the hand during steady-state walking, compared to no force, change spatiotemporal gait outcomes? To answer this question, two hypotheses have been presented to address the possibility of both adaptation and post-adaptation effects. The first hypothesis is that exposure to a haptic tensile force results in gait adaptation effects, manifested as increases in gait velocity and stride length and a decrease in double-limb support time, relative to baseline levels. The second hypothesis is that the adaptation will be maintained even after removal of the tensile force, resulting in persisting post-adaptation changes in spatiotemporal gait parameters.

## Methods

### Participants

A total of 13 healthy young adults (18–38 years old, 7 male and 6 female) participated in the study. All participants were free of any musculoskeletal, neurological, or cognitive deficits (self-report) and did not require walking aids. All participants were right-handed, thus they all held the leash in the right hand. The ethics review board of the Center for interdisciplinary research in rehabilitation of Greater Montreal (CRIR) approved the study and all participants provided their informed consent.

### Apparatus

**Fig. 1:**
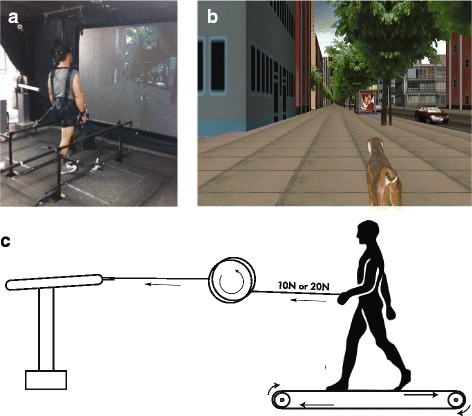
Experimental setup. (**a**) Virtual reality setup with HapticMaster, self-paced treadmill and virtual environment back-projected onto a screen in front of the treadmill. (**b**) Synchronized virtual reality scene featuring dog and city-scape. (**c**) Schematic of HapticMaster robotic arm, pulley, leash, and self-paced treadmill

Participants were fit into a safety harness and walked on a self-paced treadmill (0.8 × 1.7 m) while immersed in a virtual environment that was rear-projected onto a large screen, placed 1.5 m in front of the participant. They were given time to habituate their walking ability on the self-paced treadmill before data recording. The self-paced treadmill was custom-made on site and its motor speed was PID-controlled by an algorithm in a micro-controller using the distance signals obtained with an electro-potentiometer tethered to the back of the walking participant, as well as the first derivative of the distance (speed). The treadmill’s speed is adjusted instantaneously to keep the participant positioned at the center while walking [4]. The virtual scene’s progression was in turn controlled and synchronized with the treadmill’s speed by the CAREN-3 system (Motek BV, Netherlands). A latency between the treadmill and the CAREN system was marked at 30 ms. The virtual scene was kept contextually relevant with the physical task by featuring a sidewalk in an urban setting, with an animated dog. The dog moved in the same direction and pace as the participant (Fig. 1).

**Fig. 2:**
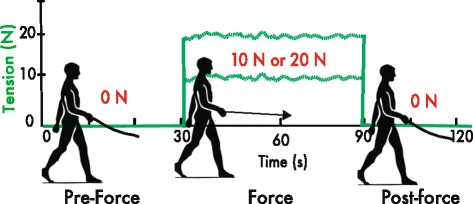
Schematic of the force step change paradigm. The green trace represents the haptic force controlled by the HapticMaster. Participants walk with 0 N during the pre-force epoch between 0 s – 30 s. Tension is then delivered to the hand during the force epoch. This tension could be either 10 or 20 N of force. The force is then released at the 90 s mark as walking continues in the post-force epoch

Participants were unexpectedly exposed to a step change in forward pulling force from 0 N to either 10 or 20 N applied to the hand. This was achieved via a leash and pulley system anchored to a force-controlled haptic robotic arm (HapticMaster, Moog BV, Netherlands) (see Figs. 1 and 2). To take into account any possible friction or stretch in the system, force levels were pre-calibrated at the hand and were transmitted through to a steel, stretch-resistant leash.

A camera-based motion capture system (six cameras, Vicon, UK) was used to record three-dimensional (3D) positions of reflective markers firmly attached to the body landmarks necessary to measure gait analysis outcomes in this study. All reflective markers were placed on the participant in accordance with the Vicon plug-in-gait^©^ 42 marker set-up. Extra markers were placed on the leash and treadmill frame. Kinematic data were recorded at a frequency of 120 Hz in the Workstation gait analysis program (Vicon, UK).

## Force step change paradigm

The paradigm used to generate a step change in force is illustrated in Fig. 2. The paradigm was divided into three distinct gait epochs; pre-force, force, and post-force (see Fig. 2). Prior to the experiment, participants were instructed to walk at a comfortable, self-selected pace throughout the paradigm. The pre-force epoch consisted of the participant walking with a slack leash (i.e. no tension) for 30 s. For analysis purposes, this epoch corresponded to baseline walking levels. An instantaneous step increase of force from 0 N to either 10 or 20 N was then transmitted to the participant’s hand via the robot-controlled leash. Participants walked with this force for 60 s. This corresponded to the force epoch. The force on the hand was then removed and participants continued to walk with a slack leash for another 30 s. This was regarded as the post-force epoch. Each force condition (10 and 20 N) was repeated twice, in a random order. The data analyzed within these epochs consisted of periods when steady-state walking was maintained at a pace consistent with daily overground walking. Hence, portions of walking data excluded from steady-state walking included the beginning and end of the trial, when participants accelerated into steady-state walking and when they started to decelerate to a stop. A period of one full gait cycle before and after the perturbation of force onset and offset was also excluded from steady-state walking.

### Data recording and analysis

Customized control software was developed to control the HapticMaster (Moog, Netherlands) robotic arm. For the beginning and ending of each walking trial, an instantaneous analog pulse was sent from the CAREN system, and was simultaneously received by both Vicon and HapticMaster systems. This enabled the synchronization of trial events across hardware platforms. This synchronization was necessary to define the critical time points of step-change force onset and offset that define pre-force, force, and post-force epochs.

Instantaneous gait velocity was derived from the 3D kinematics data and averaged over the course of a stride, using custom Matlab (Mathworks) routines. Stride length was defined by the distance between toe lifts of the same foot. Paired t-tests were conducted to compare stride lengths between the two feet. Double-limb support time was defined as the segment of the stride between the ipsilateral initial contact and the contralateral toe lift. Descriptive statistical analysis for each of the three outcomes involved percent change relative to pre-force levels. Absolute mean values were then used for statistical comparisons. We used a mixed model 2 × 3 repeated measures ANOVA taking into account force (10 and 20 N) and epoch (pre-force, force, post-force) condition levels. The covariance structure used was the variance components model, as it was found to be the most appropriate structure given the sample size [42]. Post hoc comparisons with Bonferroni adjustments were then used to investigate changes between the three epochs and two force conditions. Significance was accepted at *p* < 0.05.

## Results

### Instantaneous gait velocity

**Fig. 3:**
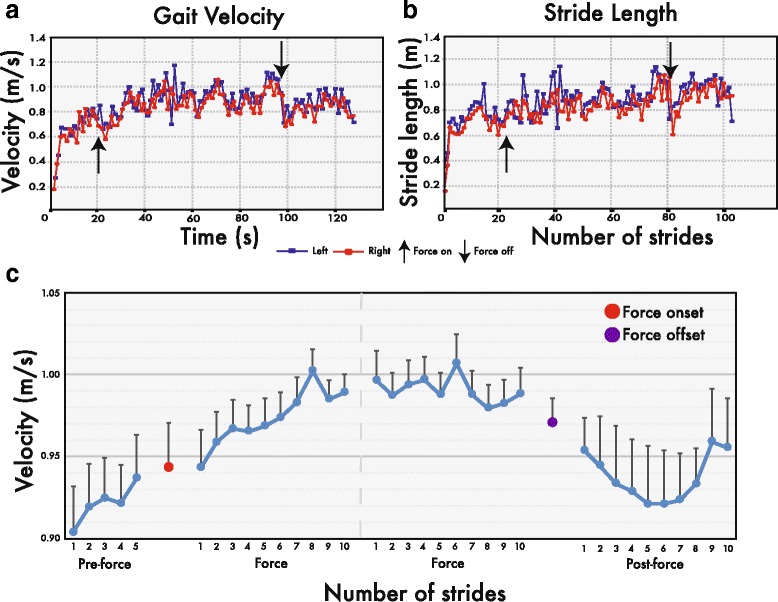
Per stride gait velocity and stride length of a single participant and average gait velocity across participants. Average instantaneous gait velocity (**a**) and stride length (**b**), per stride, for both left and right limbs of a single participant. (**c**) Average gait velocity across participants for the last five strides before (pre-force epoch) and first ten strides after 10 N force onset (force epoch), as well as the final ten strides before (force epoch) and after force offset (post-force epoch)

**Fig. 4:**
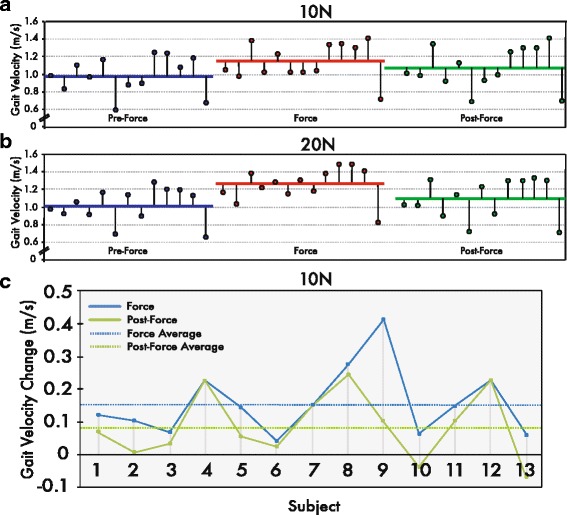
Average gait velocity per epoch and average gait velocity change for each participant. Mean instantaneous gait velocity (m/s) for each participant during the pre-force (blue), force (red) and post-force (green) epochs in the 10 N (**a**) and 20 N (**b**) conditions. The horizontal colored bars illustrate average group gait velocity. (**c**) Average gait velocity changes in the 10 N condition, for force and post-force epochs, relative to the pre-force epoch. Horizontal colored bars represent average velocity changes for force and post-force epochs across participants

Average instantaneous gait velocity for the 10 and 20 N conditions depicted in Fig. 4 reveals all 13 participants walked faster with tensile forces during the force epoch relative to the pre-force epoch. Specifically, instantaneous velocity jumped from 1.0 ± 0.2 m/s during the pre-force epoch to 1.1 ± 0.2 m/s during the force epoch for the 10 N condition and 1.0 ± 0.2 m/s to 1.2 ± 0.2 m/s for the 20 N (see blue and red horizontal traces in Fig. 4A-B). When comparing the post-force and pre-force epochs, the average gait velocity across participants tended remain above baseline levels, despite an initial decrease of 4–5 strides in response to the force offset perturbation (Fig. 3C). On average, this corresponded to 1.1 ± 0.2 m/s after exposure to the 10 N tensile force and 1.1 ± 0.2 after the 20 N tensile force (see horizontal green traces in Fig. 4A-B). A mixed model repeated measures ANOVA was used to assess the effects due to epochs and force conditions, where significant main effects were found (F(_2,24_) = 9.31, *p* < 0.01). Post hoc comparisons revealed significant increases in gait velocity in the force (*p* < 0.01) and post-force (*p* < 0.05) epochs compared to the pre-force epoch, as well as a significant increase in gait velocity from the 10 N to the 20 N conditions (*p* < 0.01).

**Fig. 5:**
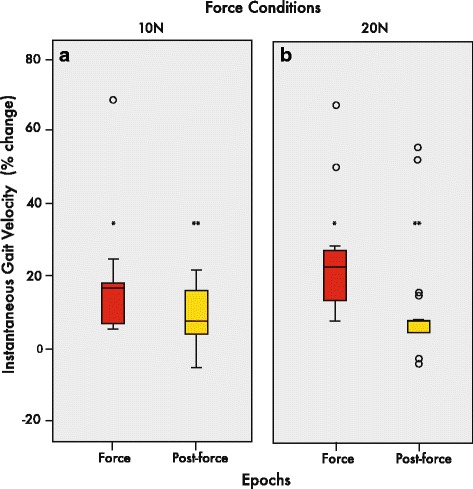
Median percent change of gait velocity. Box and whisker plots showing interquartile ranges and medians of instantaneous gait velocity percent change in 10 N (**a**) and 20 N (**b**) conditions, relative to pre-force. Gait velocity generally increased and maintained above pre-force baseline levels in 10 and 20 N conditions. **p < 0.01; *p < 0.05

The boxplots in Fig. 5A-B illustrate the interquartile median percent change of gait velocity during the force and post-force epochs relative to pre-force levels for participants in both 10 and 20 N conditions. Relative to baseline levels, all 13 participants increased their instantaneous gait velocities when walking with tension in the leash in the force epoch with 17.8% and 25.2% changes in the 10 and 20 N conditions, respectively (see Fig. 5A-B). For the post-force epoch, velocity still generally remained above baseline levels with an average increase of 9.0% and 7.0% in the 10 and 20 N conditions, respectively, relative to pre-force levels (Fig. 5A-B). A majority of the participants (11/13) demonstrated such post-adaptation changes.

### Stride length

**Fig. 6:**
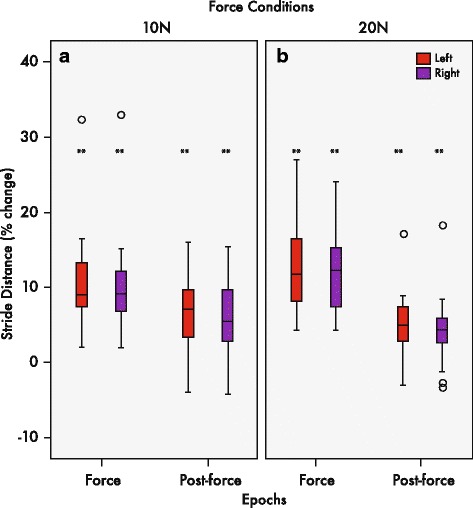
Median percent change of stride length. Box and whisker plots showing interquartile ranges and medians of stride length for the left (red) and right (purple) leg during the force and post-force epochs in the 10 N (**a**) and 20 N (**b**) conditions, relative to pre-force. Stride length increased in both epochs with respect to the baseline. **p < 0.01

An initial paired samples T-test revealed significant changes in stride length between legs (*p* < 0.05). As such, stride length was analyzed bilaterally according to the dominant leash side (right leg) and non-dominant side (left leg). The results of bilateral stride length seen in Fig. 6A-B were similar to those of instantaneous gait velocity in that all 13 participants showed changes in stride length during the force epoch relative to pre-force baseline levels in both the 10 and 20 N force conditions (Fig. 6A-B). These changes corresponded to approximately 10.6% increases for the left and right legs relative to pre-force stride lengths for the 10 N condition and 13.0% and 13.6% differences in the 20 N condition. Changes were maintained for the post-force epoch relative to pre-force levels for 10 of the 13 participants. Fig. 6A-B boxplots depict average changes of 6.5% and 6.6% in the left and right legs across all participants for the 10 N condition relative to pre-force. In the 20 N condition, post-force stride length percent change was maintained at 4.5% and 4.8% for the left and right legs. For the right leg, the mixed-model repeated measures ANOVA revealed a significant interaction of epoch and force conditions (F(_2,24_) = 6.17, *p* < 0.01). Post hoc comparisons showed significant changes in force epoch stride lengths (*p* < 0.01) compared to pre-force stride lengths in both the 10 and 20 N conditions. Only the 20 N condition showed significant changes (*p* < 0.01) in stride length between pre-force and post-force epochs. Similar post hoc comparison results were found for the left stride length (F(_2,24_) = 6.28, *p* < 0.01).

Stride length inter-stride variability for both legs was then computed with the coefficient of variation for all participants according to epochs in both 10 and 20 N conditions. During the force epoch, the variability was reduced by 1.5% on the non-dominant left leg and 1.6% on the dominant right leg for the 10 N condition relative to pre-force. Variability was also reduced by 1.2% and 1.4% on the left and right legs respectively in the 20 N condition. During the post-force, smaller reductions were seen in the variability, with 0.1% and 0.6% for the left and right legs respectively in the 10 N condition and a 1.2% increase and 1.0% decrease respectively in the 20 N condition. Despite the changes in stride length variability, a one-way ANOVA revealed no statistical significance in stride length variability across epochs and conditions for either leg (F(_5,77_) = 0.25, *p* > 0.05).

### Double-limb support time

**Fig. 7:**
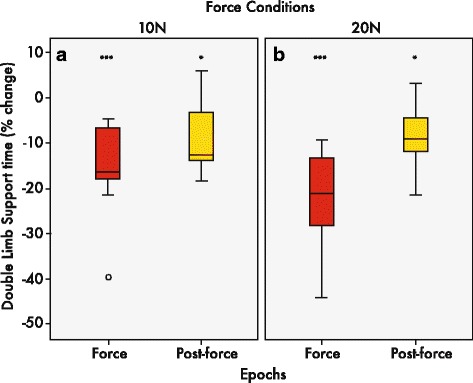
Median percent change of double-limb support time. Box and whisker plots showing interquartile ranges and medians of instantaneous double-limb support time in 10 N (**a**) and 20 N (**b**) conditions, relative to pre-force. Double-limb support times generally decreased below the pre-force baseline levels during both force and post-force epochs. ***p < 0.001; *p < 0.05

Time spent in double-limb support can be seen in Fig. 7A-B. Double-limb support times expressed as a percentage of change during the force epoch showed decreases relative to baseline pre-force levels; indicating less time spent in double-limb support in either the 10 or 20 N conditions. For example, when participants walked during the force epoch, all 13 of the participants decreased their double-limb support time. These differences correspond to an average decrease of 15.6% and 22.7% relative to pre-force values, for the 10 and 20 N conditions respectively (Fig. 7A-B). Similarly, during the post-force epoch, almost all participants showed a decrease in double-limb support times relative to the pre-force epoch. These changes in double-limb support times were maintained at 7.5% and 7.9% below baseline in the 10 and 20 N conditions, respectively, across participants. The same mixed-model ANOVA approach was used and showed once again a significant interaction between epochs and force conditions (F(_2,24_) = 3.85, *p* < 0.05). Post hoc comparisons revealed significant changes in double-limb support time in the force (*p* < 0.001) and post-force epochs (*p* < .05) relative to the pre-force epoch, in both the 10 and 20 N force conditions.

An accompanying investigation of inter-stride variability was conducted on double-limb support time. When comparing the force epoch to the pre-force, the variability was reduced by 6.5% in the 10 N condition and by 7.1% in the 20 N condition. The variability then remained lower in the post-force by 1.1% and 1.3% in 10 and 20 N conditions, respectively. A one-way ANOVA revealed a significant decrease in variability (F(_5,77_) = 2.96, *p* < 0.02), where double-limb support time during the 20 N force epoch proved significantly changed (*p* < 0.05).

## Discussion

We have investigated the extent to which haptic tensile forces applied to the hand, in the direction of walking, cause both an adaptation and a sustained post-adaptation response. As a first step, healthy young participants were recruited to establish a proof of concept by walking with and without haptic forces. In reference to the first hypothesis, walking with either 10 or 20 N of force resulted in gait adaptation effects as evidenced by changes in spatiotemporal outcomes. Specifically, both gait velocity and stride length increased, while double-limb support time decreased relative to pre-force baseline values. The second hypothesis on post-adaptation effects was also accepted, as the spatiotemporal gait changes were maintained relative to pre-force levels when the force was removed during the post-force epoch. The results suggest the evidence of both adaptation and post-adaptation effects in gait parameters due to haptic forces. It is likely that a transient, altered motor program formed during the adaptation epoch, as a consequence of the haptic tensile force, leads to lingering post-adaptation after-effects. It is not likely that the adaptation is due to a biomechanical change caused by a hauling effect on the hand. If this were the case, the adaptation effect would disappear immediately after force removal. Given that adaptations and post-adaptations were in fact present, such a sensorimotor enhancement strategy may be applied to neurological rehabilitation, such as gait enhancement post-stroke.

To this end, the spatiotemporal outcomes chosen for the study were intended to outline changes that are functionally relevant to the gait profile. Combining these outcomes can give some valid measures of both the quantity and quality of locomotion. Some fundamental findings have been highlighted in the results. The results are encouraging in that they seem to be consistent with findings from previous studies. For example, the present study provided evidence of adaptations and post-adaptations similar to those described by the broken escalator effect and the split-belt treadmill [34]. In addition, the use of a haptic force increased the capacity of dynamic stability and mobility, much like walking with a haptic strip or the instrumented cane to improve gait [28, 40].

The increase in gait velocity in this study is a clear example of an outcome exhibiting gait changes resulting from haptic forces. Namely, during the force and post-force epochs, participants on average walked faster compared to pre-force baseline velocities. This can be illustrated with gait velocity changes, relative to the pre-force epoch, of 0.16 m/s and 0.10 m/s for force and post-force epochs in the 10 N condition and 0.23 m/s and 0.10 m/s for the 20 N condition. The healthy young participants increased gait velocity by approximately 18–25% and 7–9% in force and post-force epochs, respectively in 10 and 20 N conditions (Figs. 4 and 5). Given this rather robust finding, future studies should indeed investigate such changes in the elderly and post-stroke populations.

From a clinical perspective, changes above 0.1 m/s may represent clinically meaningful changes [43–45], but it remains to be seen if post-stroke individuals are also able to increase gait velocity in excess of 0.1 m/s, either during or after force exposure, bearing in mind that they generally walk slower than healthy individuals. It is also notable that the average baseline gait velocity of 1.0 m/s in this study is slower than the normal overground walking speed, which may be attributed to walking on a self-paced treadmill in a virtual environment, as shown previously [46, 47].

Differences in stride length were seen between epochs and force conditions, and between the two legs. Bilateral stride length was a measure intended to describe the spatial and symmetrical qualities of locomotion. Evidence was found for changes in stride length between epochs. Specifically, all participants elongated their stride during the force epoch, while most maintained their stride above baseline during the post-force epoch. This was the case in both the 10 and 20 N conditions. The findings corresponded to ~ 11–14% and 5–7% changes in both legs in the force and post-force epochs (Fig. 6A-B). These changes may be translated to clinically meaningful gait outcomes in stroke and older populations [48]. Further analysis also revealed that the inter-stride variability also generally, although not significantly, decreased during the application of 10 and 20 N forces.

Less time spent in the double-limb support phase is a biomechanical consequence of increased gait speed. Per stride, this often translates to a faster, more secure dynamic posture that spends more time in the swing phase and less in stance phase, often leading to greater medial-lateral stability [49]. Given the increases in gait velocity seen in this study, reductions in double-limb support time may lend evidence for a changed gait, addressing both the quality and quantity aspects of the gait cycle. In fact, during the force epoch, the percent time of gait cycle duration of double-limb support was reduced by 16% to 23% in the force epoch in 10 and 20 N conditions. These double-limb support time reductions remained ~ 8% below pre-force levels during the post-force epoch. Further analysis of inter-stride variability showed that double-limb support time variability significantly decreased, as gait speed increased during force and post-force epochs. Again, the exciting potential from this finding is that from a clinical perspective, 0.01 s changes in double-limb support times may render clinically meaningful change, if such changes can be induced in the post-stroke population [43]. It would also be of interest if such changes translate into greater dynamic stability with the possibly of attenuating medial-lateral sway in force and post-force epochs. While investigating postural outcomes was beyond the proof of concept approach to this study, it would be interesting if similar findings can be observed in an elder chronic stroke and age-matched population.

The measures chosen were intended to test the hypotheses offered in this proof-of-principle study. As such, the reported outcomes were limited to spatiotemporal gait measures a priori. However, in light of the encouraging evidence found in gait velocity, stride length and double-limb support times, future study can focus on a more comprehensive gait analysis including kinematic and postural measures, such as joint angle changes and center of mass displacement in elder post-stroke and healthy controls. Replicating spatiotemporal findings in addition to outcomes addressing postural control and coordination in a neurological population would also provide valuable information regarding the quality and efficiency of gait changes brought on by the haptic force. Ultimately, such an analysis may help discern whether such changes in gait promote dynamic stability for functional walking in post-stroke, or other neurological conditions.

A decision was made to use 10 and 20 N force conditions in light of the eventual implementation in a post-stroke population. Lighter tensile forces may be sufficient enough to alter gait in a way to promote dynamic stability rather than hinder it. Nevertheless, it might be a limitation to use a constant tensile force rather than calibrating forces based on the participants’ body mass. Indeed, the mass was quite variable ranging from 53 to 89 kg across participants. Lastly, further study can also compare the use of visual stimuli, with particular focus on how increasing the speed of the virtual scene may affect a post-adaptation effect.

## Conclusion

This study provided evidence to suggest that the use of haptic tensile forces applied to the hand during steady-state walking elicit adaptation and post-adaptation changes to spatiotemporal components of gait in healthy young adults. The changes were evident in all measurement outcomes - instantaneous gait velocity, stride length and double-limb support time. Future studies are warranted to provide further evidence of kinematic and postural outcomes consistent with adaptation and post-adaptation effects in elderly and chronic stroke individuals. The long-term potential for using haptic tensile forces includes devising a training protocol that would evoke abiding locomotor changes, particularly in the post-adaptation phase.
